# Targeting the Hematopoietic Stem Cell Niche in β-Thalassemia and Sickle Cell Disease

**DOI:** 10.3390/ph15050592

**Published:** 2022-05-11

**Authors:** Annamaria Aprile, Silvia Sighinolfi, Laura Raggi, Giuliana Ferrari

**Affiliations:** 1 San Raffaele-Telethon Institute for Gene Therapy (SR-TIGET), IRCCS San Raffaele Scientific Institute, 20132 Milan, Italy; sighinolfi.silvia@hsr.it (S.S.); raggi.laura@hsr.it (L.R.); 2 Vita-Salute San Raffaele University, 20132 Milan, Italy; 3 University of Milano Bicocca, 20126 Milan, Italy

**Keywords:** β-thalassemia, sickle cell disease, bone marrow niche, hematopoietic stem cells

## Abstract

In the last decade, research on pathophysiology and therapeutic solutions for β-thalassemia (BThal) and sickle cell disease (SCD) has been mostly focused on the primary erythroid defect, thus neglecting the study of hematopoietic stem cells (HSCs) and bone marrow (BM) microenvironment. The quality and engraftment of HSCs depend on the BM microenvironment, influencing the outcome of HSC transplantation (HSCT) both in allogeneic and in autologous gene therapy settings. In BThal and SCD, the consequences of severe anemia alter erythropoiesis and cause chronic stress in different organs, including the BM. Here, we discuss the recent findings that highlighted multiple alterations of the BM niche in BThal and SCD. We point out the importance of improving our understanding of HSC biology, the status of the BM niche, and their functional crosstalk in these disorders towards the novel concept of combined therapies by not only targeting the genetic defect, but also key players of the HSC–niche interaction in order to improve the clinical outcomes of transplantation.

## 1. Introduction

Hemoglobinopathies are inherited disorders affecting hemoglobin (Hb) production, estimated to be the most common monogenic diseases worldwide [1]. They include deletions or point mutations in α- or β-globin genes encoding for Hb chains, resulting in hemolytic anemia. Mutations can cause abnormalities in the amount of Hb, leading to thalassemia syndromes, such as β-thalassemia (BThal), or in the Hb structure, as in sickle cell disease (SCD). The highest prevalence of both diseases has initially been recorded in the Mediterranean area and Africa; however, because of migrations, they represent a global health problem nowadays. The severe form of BThal requires lifelong transfusions associated with iron chelation therapy [2]. As a major consequence of the disease and treatment, iron overload (IO) occurs in most cases, despite iron chelation, causing chronic organ damage. SCD is characterized by anemia and vaso-occlusive crises (VOCs), which can cause ischemic and oxidative organ damage [3]. Allogeneic hematopoietic stem cell transplantation (HSCT) from healthy donors is the only curative option for both BThal and SCD patients [4,5,6,7]. Gene therapy by gene addition or gene editing strategies in autologous hematopoietic stem cells (HSCs) are promising curative alternatives for patients lacking a suitable donor [8,9,10,11,12,13]. Cases of graft failure have been reported, but causes have not been deeply investigated and can include an impaired HSC function (autologous setting) and/or a defective supporting activity of the bone marrow (BM) niche (autologous and allogeneic settings), worsened by age and disease progression.

HSCs are regulated by signals from the BM microenvironment, termed niche, which tune hematopoiesis. Stromal and hematopoietic BM cells provide support to HSC activity and include mesenchymal stromal cells (MSCs), endothelial cells (ECs), megakaryocytes (MKs), and many other hematopoietic cells representing the differentiated progeny of HSCs [14,15]. Niche composition is dynamic and changes in response to perturbed hematopoiesis or regeneration following myeloablation and transplantation [16]. Many studies unraveled its regulation upon stress or in malignancies [17], but little is known about HSC–niche interactions in hematological-inherited disorders.

Over decades, research on hemoglobinopathies has been mostly focused on the primary erythroid defect, leaving the study of the HSC and the BM microenvironment almost completely unexplored. Since the quality and the engraftment of HSCs depend on the BM microenvironment, niche–HSC crosstalk is important for the outcome of allogeneic and especially autologous gene therapy HSCT, where both HSCs and BM niche are under stress.

Accumulating evidence highlights that, despite differences in etiology, in both BThal and SCD, the consequences of severe anemia alter BM erythropoiesis and cause chronic stress in different organs, including in the BM. In this view, recent findings challenged the paradigm of BThal and SCD, as disorders confined to erythropoiesis [18] and highlighted multiple alterations in the BM niche, paving the way towards potential combined therapies targeting not only the genetic defect, but also HSCs and the BM microenvironment, with the idea of “*Protecting the seed, fertilizing the soil*” [19].

In the present review, we summarize the state-of-the-art about HSCs and BM niche defects in BThal and SCD, and we discuss potential therapeutic solutions to ameliorate HSC and BM microenvironments in order to improve the clinical outcome of HSCT. We point out the importance of having a better understanding on HSC biology, the status of the BM niche, and their functional crosstalk towards the novel concept of targeting the BM niche.

## 2. Hemoglobinopathies

Hemoglobinopathies are a group of widespread inherited blood disorders primarily affecting red blood cells (RBCs), caused by mutations in the genes encoding for the chains of Hb. Hb is a tetramer composed of four polypeptide chains, each carrying a heme prosthetic group containing an iron molecule. During development, different combinations of globin chains are assembled. The first Hb switching event occurs with the transition of the site of erythropoiesis from the yolk sac to the fetal liver from the production of embryonic Hb to fetal Hb (HbF, α2γ2). The second switch in humans occurs perinatally with the decline in HbF synthesis, coupled with the increase in the adult form of Hb (HbA, α2β2). α- and β-chains are tightly regulated to ensure balanced synthesis. Hemoglobinopathies are divided into two main groups: thalassemia syndromes with quantitative defects leading to reduced levels of one type of globin chain, and structural Hb variants, such as SCD, with qualitative defects causing a change in the structure of the Hb molecule. The highest prevalence of both diseases has been recorded in the Mediterranean countries, Middle East, Indian subcontinent, Southeast Asia, and Africa. The distribution of inherited Hb variants in specific regions is attributed to the natural selection of heterozygote carriers for resistance against *Plasmodium falciparum* malaria [20]. Through migration flows, hemoglobinopathies have spread in Northern Europe, North America, and Australia, evolving into a global health issue.

### 2.1. Thalassemia

Thalassemias are autosomal recessive diseases in which mutations occur in either α- or β-globin genes, resulting in phenotypes with different severities of anemia due to the imbalanced production of globin chains of adult HbA [21]. It has been estimated that about 1.5% of the world population is a carrier of thalassemic mutations and more than 60,000 symptomatic individuals are born every year [22].

α-thalassemia is characterized by the deficient synthesis of α-globin chains caused by deletions within the α-globin gene cluster on human chromosome 16 [23].

BThal is caused by mutations in the β-globin gene on human chromosome 11, leading to reduced (β+) or absent (β0) synthesis of β-globin chains [24]. Based on the severity of the clinical phenotype, patients with BThal have been traditionally classified as major (β0β0 or β0β+), intermedia (β+β+), or minor (heterozygote carrier) [25]. However, more recently, BThal patients have been divided based on transfusion therapy requirements into transfusion-dependent thalassemia (TDT) or non-transfusion-dependent thalassemia (NTDT). TDT patients are not capable of producing sufficient Hb and undergo lifelong transfusion treatments for survival, while NTDT ones can still require transfusions occasionally and not for their entire lifetime [2].

More than 300 BThal alleles have been identified, most of which result in a single-nucleotide substitution in the β-globin gene or flanking regions, as well as small deletions or insertions within the gene or upstream regulatory elements may occur [25]. At the molecular level, BThal mutations lead to an imbalance of α/β-globin chains, and an accumulation of the free α-globins which form toxic aggregates. Free Hb and heme catalyze the formation of reactive oxygen species (ROS), leading to oxidative damage and hemolysis of circulating RBCs, as well as premature apoptosis of erythroid precursors in the BM. BThal disease manifestations result in chronic severe anemia which stimulates the production of erythropoietin (EPO) with a consequent intensive but ineffective expansion of the BM erythroid compartment, leading to ineffective erythropoiesis (IE), altered BM homeostasis, and stress signals [21]. In addition, erythroid activity increases in extramedullary hematopoietic sites, causing splenomegaly and hepatomegaly.

In BThal multi-organ complications secondary to the primary genetic defects occur, such as endocrinopathies, including hypogonadism, hypothyroidism, and hypoparathyroidism, as well as impaired bone metabolism. Indeed, bone disease, such as osteopenia and osteoporosis, is a common complication of BThal patients [26,27]. Osteoporosis is characterized by a significant decrease in bone mineral density (BMD), associated with a high prevalence of fractures. The causes of bone loss are controversial [28] and a possible molecular mechanism foresees the decrease in the osteoprotegerin (OPG)/receptor activator of nuclear factor kappa-β ligand (RANKL) ratio, resulting in increased expression of RANKL by stromal cells or osteoblasts (OBs), which contributes to enhanced bone resorption by osteoclasts (OCs) [29]. However, the pathophysiology of bone loss is still under investigated in BThal patients since bone disease retains high morbidity despite therapies.

Finally, IE results in increased iron absorption and primary IO mediated by the decrease in hepatic hormone hepcidin levels. Moreover, transfusional iron intake saturates transferrin receptor capacity, leading to secondary IO, causing damage especially in the liver, heart, and endocrine organs [2]. Despite the improvement in iron chelation therapies, IO remains one of the most relevant clinical complications in BThal patients.

### 2.2. Sickle Cell Disease

SCD is an autosomal recessive inherited blood disorder that affects millions of people worldwide [1].

SCD is caused by a single-nucleotide mutation of the β-globin gene, resulting in the production of hemoglobin S (HbS), which induces RBCs to become rigid, sticky, and misshapen. HbS consists of an A to T transversion, leading to a substitution of a valine for glutamic acid at position 6 in the β-globin chain [30]. The most common type of SCD is the severe form, in which people inherit two sickle cell genes. Other forms of SCD include compound heterozygous conditions, such as HbC with HbS (HbSC) and HbS with BThal (HbS/β0-thalassemia or HbS/β+-thalassemia) [3].

HbS has a lower oxygen affinity compared to HbA and this condition increases HbS polymerization, promoting the formation of sickle-shaped RBCs (SS-RBCs) [31]. HbS polymerization induces cellular rigidity and changes the shape and physical properties of RBCs, impairing their rheology and survival [32]. Abnormal SS-RBCs cause hemolytic anemia, cell adhesion, vasoconstriction, and vaso-occlusion in small vessels.

Clinically, the major symptom of SCD is a pain crisis due to the vaso-occlusion of tiny blood vessels to the chest, abdomen and joints, resulting in VOCs. Several factors trigger VOCs, including the endothelium which becomes activated by SS-RBCs, recruitment of adherent leukocytes, activation of neutrophils, interactions of RBCs with adherent neutrophils, and ischemia due to the obstruction, thus creating a feedback loop of worsening endothelial activation. Moreover, the damaged SS-RBCs and activated ECs can produce a proinflammatory environment that is exacerbated during episodes of crisis. They may lead to the production of ROS by the ECs and oxidant-dependent activation of the transcription factor NF-κB, causing inflammatory vasculopathy and vasospasm [33].

Skeletal abnormalities are often associated with SCD [34]. Sickle bone shows reduced BMD, widening of the marrow cavity, and thinning of the cortical bone due to erythroid hyperplasia associated with the disease. More than half of SCD patients have low BMD, closely resembling the features of osteoporotic bone and nearly 30% of adult patients reported multiple fractures due to low impact trauma. Due to vulnerability to infections, osteomyelitis occurs frequently in SCD patients and is caused by several types of germs that enter the bone, leading to osteonecrosis, impaired growth in children, and septic arthritis.

### 2.3. Therapeutic Options for Hemoglobinopathies

Chronic management for BThal and SCD includes blood transfusion, administration of hydroxyurea, and iron chelation therapy. A better understanding of the pathogenesis and improved treatments for hemoglobinopathies have dramatically ameliorated the life expectancy of patients.

The mainstay treatment for severe BThal patients is the association of regular blood transfusion and iron chelation therapies. Starting in the first few years of life, they suppress IE, thus limiting downstream pathophysiological complications, such as heart disease, bone and endocrine abnormalities, and increasing overall survival of TDT. Iron chelation can reduce systemic and hepatic iron burden, both in TDT and in NTDT, decreasing the risk of complications related to IO. However, these therapies are often ineffective due to repeated transfusions and inadequate patient compliance.

SCD patients need single acute transfusions by replacing SS-RBCs for immediate benefit, while chronic transfusions help in preventing long-term complications. Regularly transfused SCD patients require iron chelation treatments too. Hydroxyurea is a myelosuppressive agent which is used to prevent painful episodes in SCD. Current evidence suggests that it acts by inducing HbF, which in turn inhibits intracellular HbS polymerization [35].

The only definitive curative option for both BThal and SCD patients is represented by the replacement of diseased RBCs with those differentiating from transplanted normal HSCs [4,6,7,36]. Allogeneic HSCT from normal donors is available to less than 20% of patients and it is limited by the difficulty of finding suitably matched donors and the risk of graft rejection [5]. High mortality in patients who underwent transplants was observed with increased patient age; thus, current recommendations offer allogeneic HSCT to patients younger than 14 years old who have a suitable HLA-matched donor.

Over the last few decades, promising therapeutic approaches have been developed to achieve definitive correction of the erythroid defect by ex vivo gene therapy through both gene addition and gene editing strategies [37]. Gene therapy approach consists of autologous transplantation of genetically modified hematopoietic stem and progenitor cells (HSPCs) using lentiviral vectors expressing a globin gene (β-globin or fetal γ-globin) under the control of globin transcriptional regulatory elements. Recent gene therapy clinical trials for BThal and SCD have shown promising results. LentiGlobin^TM^ BB305 medicinal product (commercial name Zyntheglo) consists of autologous CD34^+^ HSPCs transduced with BB305 lentiviral vector that encodes for anti-sickling hemoglobin (HbA^T87Q^) and showed safety and efficacy in severe SCD and TDT patients with a reduction in transfusion requirements up to transfusion independence in the vast majority of patients [9,11,12,38]. Furthermore, in the TIGET-BTHAL clinical trial, the primary endpoints of safety and efficacy were achieved. Decreased transfusion requirement in adult and pediatric TDT patients and complete independence from transfusions in three of four evaluated children were obtained by intrabone administration of autologous HSPCs transduced by the GLOBE lentiviral vector [10]. A different approach employed a lentiviral vector expressing a small hairpin RNA targeting the γ-globin repressor BCL11A to increase HbF concentration with an anti-sickling effect. It was used in patients with severe SCD and showed promising initial results [39]. Regarding gene editing strategies, they are based on the direct correction of genetic mutation or disruption of specific DNA sequences in the genome using nucleases. Ongoing clinical trials in TDT and SCD patients are testing the safety and efficacy of HSPCs edited with CRISPR-Cas9 (CTX001 drug product) [13] or with ZFNs (ST-400 drug product). In both cases, targeted nuclease disruption of the repressor BCL11A gene led to γ-globin reactivation with HbF synthesis. Extended follow-up will define the long-term effects of gene therapy approaches.

Recently, other experimental therapeutic approaches are under investigation. A novel strategy aimed to ameliorate IE consists in Luspatercept administration, although the exact mechanism of action is not fully understood. Luspatercept binds to transforming growth factor (TGF) β superfamily ligands, enhances late-stage erythropoiesis by blocking SMAD2/3 signaling, and reduces RBC transfusion requirements in TDT with a proportion of patients achieving transfusion independence [40]. Mini-hepcidins (i.e., short peptides that mimic the activity of endogenous hepcidin) [41] and TMPRSS6 inhibitors [42] showed significant improvements in IE, anemia, and IO in mouse models. Moreover, phase I clinical studies of Vamifeport, the first oral ferroportin inhibitor, showed efficacy in reducing cellular iron efflux and in ameliorating IE [43].

Overall, HSCT remains the only definitive cure for hemoglobinopathies. Despite decades of allogeneic HSCT experience and promising results following gene therapy clinical trials for BThal and SCD, cases of graft failure have been reported. The causes need further investigation, but it can include a negative role of stressed BM microenvironment worsened by age and disease progression [10,37]. In gene therapy, the clinical outcome depends on different factors, such as the source of HSCs, the efficiency of transduction, and the status of the BM microenvironment [44]. Furthermore, a recent halt in SCD gene therapy trials, due to the development of myeloid neoplasms [45], raises the need for a better characterization of the BM microenvironment in genetic Hb disorders. Studies on the BM milieu in BThal and SCD suggest that the BM niche could have an impact on HSC function, thus potentially affecting the HSCT clinical outcome.

## 3. HSC and the BM Niche

The adult hematopoietic system is maintained by HSCs, which are able to self-renew and give rise to progenitor cells that proliferate and differentiate into all the mature blood cells. HSCs are essential to replenish the hematopoietic system after transplantation and upon exposure to stressors, such as oxidative stress and inflammatory signals [17].

HSCs reside in a specialized microenvironment within the BM, termed niche. At a steady state, HSCs are maintained quiescent to avoid the exhaustion of the stem cell pool, but they can rapidly exit from dormancy in response to stress conditions. HSC behaviour is governed by the complex interactions with different cellular components of the BM niche, soluble factors, and physical cues [15]. High-resolution imaging studies in mice revealed that HSC niche include endosteal niches, near the bone surface, and vascular niches, which can be further divided based on the proximity to arterioles or sinusoids.

### 3.1. HSC Regulation by the BM Niche

Over the last two decades, the regulation of the BM niche at steady state has been extensively studied thanks to technical advances in mouse genetics and imaging technologies [46]. BM populations are intimately connected with each other; alterations of specific niche cells or depletions of specific factors could negatively impact other niche components, thus affecting HSC function. Several non-hematopoietic and hematopoietic cell types have been pointed out.

**Stromal BM cells.** BM stromal components, such as osteolineage cells, MSCs, and ECs, provide physical support and control HSC homeostasis.

Osteolineage cells, lining the endosteum, were the first population to be associated with HSC regulation [47,48]. OBs are activated by the parathyroid hormone (PTH), and PTH-stimulated OBs are increased in number and produce high levels of the Notch ligand Jagged-1 (JAG1), which in turn supports HSC expansion [49].

Osteolineage cells produce many cytokines, including osteopontin (OPN), C-X-C motif chemokine ligand 12 (CXCL12) (also known as stromal derived factor 1 (SDF1)), stem cell factor (SCF), thrombopoietin (TPO), and angiopoietin1 (ANGPT1). OB-derived OPN was reported to control HSC homing and engraftment, as well as suppress the proliferation of HSCs [50]. However, recent evidence suggests that osteolineage cells do not directly regulate HSCs, since other populations are the main functional sources of CXCL12, SCF, TPO, and ANGPT1 [51,52,53,54]. Although these studies raised doubts about the essential role of OBs for HSC maintenance, recent in vivo live imaging suggests that HSC localization and function are tightly dependent on bone turnover [55].

MSCs are a rare population in the BM located around the blood vessels, which can self-renew and differentiate into bone, fat, and cartilage. CD146 marks human MSCs that generate colony-forming unit fibroblasts (CFU-Fs) in vitro and heterotopic ossicles in vivo [56,57]. In mice, different MSC populations were identified using transgenic models, including Nestin^+^ perivascular cells, CXCL12-abundant reticular (CAR) cells, leptin receptor (LEPR)^+^ cells, and PDGFRα^+^ Sca1^+^ (PαS) cells [51,58,59,60]. However, it is now widely recognised that there is overlap among them. MSCs modulate HSC activity by direct interaction or through the secretion of soluble factors. Among these, N-cadherin mediates the adhesion of human CD34^+^ HSPCs to MSCs, preserving their repopulating ability in a co-culture assay [61]. Of note, N-cadherin^+^ stromal progenitors preserve HSCs upon chemotherapy insult [62]. MSCs mainly control HSCs through the release of SCF and CXCL12 along with other regulatory factors, such as ANGPT1, OPN, and interleukin 7 (IL-7). Moreover, MSCs protect HSCs from stress signals, such as oxidative stress and inflammation. High ROS levels affect HSC quiescence and function, and MSCs act as ROS scavengers, preventing myeloablation-induced oxidative damage in HSCs [63].

Adipocytes that arise from MSCs are negative regulators of HSC maintenance. The number of BM adipocytes inversely correlates with HSC content, and adipogenesis inhibition accelerates HSC engraftment after transplantation or chemotherapy [64]. By contrast, adipocyte progenitors were found to sustain HSC regeneration upon chemotherapy by secreting SCF [65]. Further investigation is required to clarify their regulatory activity.

BM ECs, lining the surface of blood vessels, control vascular integrity, which in turn affects HSC trafficking and function. Two different EC types can be distinguished based on their localization and the differential expression of surface markers, i.e., arteriolar and sinusoidal ECs. The permeability of blood vessels regulates ROS levels in adjacent HSCs and niche populations, and less permeable arterioles maintain HSCs in a quiescent state with low ROS levels, whereas leaky sinusoids increase ROS levels in HSCs, thus leading to their activation and mobilization into the circulation [66]. ECs provide soluble factors that promote HSC self-renewal and hematopoietic regeneration after injury [67,68]. A loss of endothelial CXCL12 or SCF causes HSC depletion [51,69].

The sympathetic nervous system, which innervates both the bone and the BM, also regulates HSC function. Nerve fibers form a network with perivascular stromal cells, controlling HSC mobilization into the bloodstream and stimulating the hematopoietic recovery after genotoxic stress [70,71]. Non-myelinating Schwann cells that wrap sympathetic nerves contribute to HSC quiescence through activation of TGFβ and SMAD signaling [72].

**Hematopoietic BM cells.** Moreover, terminally differentiated cells, including MKs, macrophages (Mφs), neutrophils, and regulatory T cells (Tregs), play a key role in the BM niche.

Initial studies showed that HSCs are located close to MKs in the BM in a non-random fashion, and MK depletion increases the size of the HSC pool [73]. At a steady state, MKs directly maintain HSC quiescence by secreting specific factors, such as CXCL4 and TGFβ [73,74]. However, under chemotherapeutic stress conditions, MKs promote HSC expansion through fibroblast growth factor 1 (FGF1) signalling [74]. In addition, MKs regulate HSC function indirectly through the interaction with bone cells [75,76]. Furthermore, after myeloablative irradiation, MKs support OB survival by promoting HSC engraftment [77].

Mφs are an heterogenous population with phagocytic activity present in various tissues. In response to different environmental signals, they can polarize to M1 (classically activated or pro-inflammatory) or M2 (alternatively activated or anti-inflammatory) phenotypes, protecting from infections and promoting tissue repair and regeneration, respectively. BM Mφs directly regulate HSC quiescence and self-renewal [78,79,80]. M2 Mφs induce HSC self-renewal, whereas M1 Mφs exert an opposite role in preserving HSC repopulating potential [81]. In addition, BM Mφs indirectly control HSC location by inducing the expression of HSC retention factors by MSCs, thus regulating the egress of HSCs into the circulation through neutrophil clearance [82].

Neutrophils control HSC retention by acting on osteolineage cells [83] and play a key role in the regeneration of the BM niche after transplantation [84]. Whether neutrophils can directly regulate HSC function remains poorly understood.

Tregs modulate HSCs by cytokine secretion and host immune regulation [85,86].

### 3.2. HSC Regulation by Stress Signals

Hematopoiesis can be challenged by different sources of stress, such as oxidative stress, variations in the partial pressure of oxygen, iron levels, and inflammatory signals. Quiescent HSCs reside in a hypoxic BM niche that promotes glycolytic metabolism over oxidative phosphorylation and protects HSCs from oxidative stress. Stress signals alter HSC function by impairing their self-renewal and regeneration capacity [16].

Oxidative stress is the result of intracellular ROS accumulation which causes oxidative damage of lipids, DNA, and proteins in stem cells [87]. ROS tightly regulates HSC function through direct modulation of redox-sensitive transcription factors [88] and HSCs maintain a low basal level of ROS, which preserves stem cell quiescence. In contrast, a physiological increase in ROS leads to cell proliferation and differentiation [89]. Moreover, the impairment of antioxidant defense mechanisms reduces HSC quiescence and repopulating ability [90].

Iron is an essential element required for many cellular functions; however, when in excess, it can cause uncontrolled production of ROS through the Fenton reaction. Moreover, heme, an iron-containing porphyrin, constitutes 95% of the total iron in humans and its degradation mediated by heme oxygenase 1 releases iron with further ROS production [91]. HSCs can uptake and store iron, and fluctuation of iron levels can regulate HSC function. Initial studies in an iron-overloaded mouse model revealed a ROS-mediated reduction in HSPC number and repopulating ability [92]. Moreover, intracellular IO in HSCs promotes oxidative stress, leading to their dysfunction and exhaustion [93]. Vice versa, treatments with iron chelators in vivo and in vitro restore HSPC quiescence and self-renewal [94]. Additionally, iron deficiency seems to negatively affect HSC function [95]. However, it remains to be explored how intracellular iron levels affect the metabolic and transcriptional programs underlying HSC function and fate.

Recent advances have highlighted the critical role of inflammation as a source of stress, affecting HSC fate. The expression of Toll-like receptors (TLRs) on HSC surfaces allows them to directly sense pathogen-derived products. HSCs proliferate, lose self-renewal capacity, and rapidly differentiate in response to many inflammatory signals, such as interferons (IFNs), tumor necrosis factors (TNFs), IL-1, granulocyte-colony stimulating factor (G-CSF), and damage-associated molecular patterns (DAMPs), in order to replenish myeloid cells [96]. However, chronic exposure to inflammatory cytokines can injure HSCs [97], and repeated activation of HSCs in response to inflammatory stimuli can cause DNA damage mediated by high ROS levels [98]. Furthermore, infections trigger hematopoiesis by acting on HSC cycling properties and long-term function [99] or on BM niche populations, such as ECs, MSCs, osteolineage, and immune cells [100,101,102].

Thus, given the complexity and heterogeneity of the BM niche, it is conceivable that stress signals exert their effect on HSCs, both directly and indirectly, by acting on niche populations.

### 3.3. The HSC Niche in Aging and Disease

Recently, the effects of stressed, aged, and malignant hematopoiesis on HSCs, as well as the BM microenvironment, have gained increasing attention [17].

During physiological aging, HSC function declines with alterations in immune responses, contributing to higher susceptibility to infections, autoimmunity, anemia, and myeloproliferative diseases. The absolute numbers of HSCs increase in aged mice, but their regenerative potential is reduced with myeloid-biased differentiation and enhanced mobilization into the circulation [103]. Aged HSCs localize far from the endosteum and close to sinusoidal ECs [104], suggesting that altered HSC distribution is a hallmark of aging. Aging leads to remodeling of the BM niche with a loss of innervation of BM arterioles [105], and also skews MSCs differentiation with reduced bone formation and increased adipogenesis.

The BM niches have been recognized as playing a key role in the pathogenesis and chemoresistance of hematological malignancies [106]. On the one hand, BM niche populations can facilitate the survival and the expansion of mutant hematopoietic cells, contributing to malignancy progression and providing protection of malignant cells from chemotherapy. On the other hand, malignant hematopoiesis can remodel the HSC niche [107,108,109].

Conversely, the composition and properties of the BM niche in non-malignant hematopoietic disorders affecting the differentiated progeny of HSCs is still under investigated. Few reports highlighted BM defects in severe combined immunodeficiencies (SCID), such as adenosine deaminase-SCID (ADA-SCID) with myeloid dysplasia, marrow hypocellularity, and a reduced frequency of HSCs [110,111]. In patients affected by chronic granulomatous disease (CGD), hematopoiesis was shown to be dysregulated, and a reduced proportion of HSCs was reported as a direct consequence of the inflammatory state associated with the disease [112]. In addition, HSPCs from patients affected by Wiskott–Aldrich syndrome displayed altered cytoskeleton function, impaired migratory, and homing capacity [113].

Thus, a better characterization of the features of the BM microenvironment and HSCs in hematological-inherited disorders, including BThal and SCD, is fundamental to assess potential defects and to improve therapeutic approaches based on allogeneic HSCT and autologous gene therapy [44].

## 4. The HSC Niche in BThal and SCD

The HSC BM niche is still poorly investigated in hemoglobinopathies. The status of HSCs and BM niche, indeed, should be considered in the context of allogeneic HSCT to ensure a sustained engraftment of donor cells and especially in recent autologous gene therapy settings, to develop optimized protocols for increasing the efficacy and safety of ex-vivo genetic manipulation [44]. The reduced number of HSCs available for collection and the impaired engraftment potential of HSCs in BThal and SCD mouse models [114,115] highlighted the need for a better characterization of the primitive HSC compartment and BM niche features to develop targeting strategies for improving the outcome of allogeneic and autologous HSCT.

To study HSC niche in BThal and SCD, mouse strains recapitulating the main features of the human diseases were exploited [116]. An investigation of BThal HSCs and BM niche was performed in the Hbb^th3/+^ (th3) murine model. th3 mice lack both the adult β^maj^- and β^min^-globin genes [117]. Although homozygous knockout mice are not viable, heterozygotes survive with features of severe BThal intermedia, including reduced RBC and Hb concentrations, microcytosis, reduced hematocrit and elevated reticulocytes, IE, splenomegaly, bone malformation, and IO in multiple tissues. The studied mouse models for SCD include the transgenic SAD, Berkley, and Townes strains [116]. They were generated by co-expression of the human α2-globin gene and a modified βS-globin gene, both linked to the β-globin locus regulatory region. The SAD mouse incorporates the βS variant with additional mutations known to enhance the severity of the sickle phenotype into a BThal background [118], whereas the Berkley and Townes transgenic strains carry the genes for human α- and βS-globin on a genetic background deficient for the murine β-globin genes and incorporate a YAC containing one or both γ-globin gene sequences to avert the gestational lethality [119,120]. These models reproduce in vivo sickling of RBCs, hypoxia, and severe anemia with reduced hematocrit and increased reticulocytes, systemic microvascular occlusions, hemolytic and renal complications, splenomegaly, and IE of human SCD. Patient-derived samples were studied for the immunophenotypic characterization of HSCs and BM microenvironment to validate findings obtained in mice and to model in vitro the discovered alterations.

Here, we review the state of the art about the alterations of HSCs and BM niche populations in BThal and SCD.

### 4.1. HSCs

**BThal.** A recent study by Aprile et al. has provided the first demonstration of impaired HSC function caused by an altered BM niche in BThal [114]. The authors demonstrated that th3 mice have a decreased number of HSCs, as compared to wild-type (wt) controls. Cell cycle analysis revealed a loss of quiescence with a lower frequency of HSCs in the G0 phase and an increased cycling rate with a higher fraction of cells accumulated in the S phase. These data were corroborated by RNA-seq experiments performed on sorted th3 HSCs, revealing a positive enrichment of cell-cycle-associated categories, a upregulation of genes involved in DNA damage, cellular responses to stress, and a downregulation of stemness genes (including *Cdkn1c*, *Runx1l1*, *Fgd5*, and *Hes1*), thus highlighting the increased replication stress and impaired self-renewal ability of HSCs in BThal. To evaluate the functional activity of BThal HSCs, they performed long-term in vivo transplant experiments and they observed a competitive disadvantage of th3 HSCs compared to wt ones when transplanted into th3 mice. Notably, transplantation into wt recipients rescued the long-term repopulating capacity of th3 HSCs, suggesting that the wt BM microenvironment had a corrective role in restoring HSC functions. Secondary transplants into wt animals showed that th3 HSCs recovered their reconstitution capacity with complete normalization of the quiescent state. On the contrary, th3 HSCs in BThal recipients underwent exhaustion over time. These results suggested that impaired HSC self-renewal and quiescence in BThal are not intrinsic defects, but their behavior is affected by prolonged residence in an altered BM microenvironment, which is progressively worsened by the disease.

In line with these findings, patients affected by TDT showed reduced quiescence of CD34^+^CD38^-^ primitive HSPCs [114]. Moreover, the gene expression profile of patients’ CD34^+^ cells revealed an upregulation of genes associated with stress stimuli and DNA damage, thus indicating an impairment of HSPCs also in the human disease. Consistently, Hua and colleagues published a reduced frequency of Lin^−^ CD10^−^ CD34^+^ CD38^-\low^CD45RA^−^ CD90^+^ HSCs in the CD34^+^ cell compartment of BThal pediatric patients [121]. Moreover, and most importantly, RNA-seq analysis of BThal patients’ HSCs showed increased proliferation and reduced stemness [122].

Overall, this evidence highlighted the importance of the BM microenvironment in preserving HSC fitness in the BThal context.

**SCD.** Studies in the murine models of SCD revealed defects in SCD hematopoiesis and HSCs. In SCD, the BM environment is highly enriched for ROS, mainly generated by SS-RBCs and the activated endothelium. By examining the effects of oxidative stress on SCD HSCs, Javazon et al. showed that SCD BM has a decreased colony forming unit potential and a reduced number of Lineage^-^ Sca1^+^ cKit^+^ (LSK) HSPCs [123]. Cell cycle analyses revealed that fewer LSK cells were in the G0 phase, and a significant increase in lipid peroxidation and ROS in SCD LSKs was detected. HSPCs from SCD mice showed an impaired engraftment potential, which is partially restored by n-acetyl cysteine (NAC) antioxidant treatment of LSK cells before transplantation, thus suggesting that an altered redox environment in SCD affects HSC function.

The results of reduced clonogenic potential of SCD HSPCs are paralleled by the increased mobilization of multipotent cells in both mice and humans affected by SCD. A hematopoietic compensatory mechanism was described in SCD, consisting in the mobilization of progenitor cells from the BM to the peripheral blood and their subsequent uptake into the splenic extramedullary hematopoietic site in response to the erythropoietic stress [124]. The spleen of SCD mice indeed contains significantly increased numbers of cycling erythroid colony-forming cells, indicating the strong proliferative pressure on the erythroid lineage.

Alterations in SCD HSCs were reported by Tang et al., who showed a decrease in HSC frequency, increased DNA damage, and an accumulation of ROS in HSCs from SCD mice, associated with the reduced hematopoietic supportive ability of MSCs [115].

Recently, Hua et al. showed a lower proportion of Lin^−^ CD10^−^ CD34^+^ CD38^-\low^CD45RA^-^ CD90^+^ HSCs in the CD34^+^ cell compartment of SCD pediatric patients, along with an increased frequency of CD34^+^ CD10^+^ lymphoid progenitor cells [121], suggesting hematopoietic defects also in the human disease. Moreover, the characterization of circulating hematopoietic populations from adult and pediatric SCD patients confirmed an increase in CD34^bright^ HSPCs and the mobilization of primitive HSCs in the peripheral blood as compared to healthy controls [125].

### 4.2. The Stromal Niche

**BThal**. To explain the defects in the transplantation outcome of th3 HSCs into a BThal BM niche, Aprile et al. focused their attention on the interactions between HSCs and stromal cells, such as osteolineage cells and MSCs in the BM of BThal mice [114]. Bone disease is a common and severe complication of BThal, resulting from hormonal deficiency, BM expansion, and iron toxicity. Consistent with the common finding of osteoporosis and hypoparathyroidism in BThal patients [27], data in th3 mice confirmed a reduced BMD [126] caused by decreased OB activity and low levels of circulating PTH [114]. Moreover, the authors showed reduced levels of key niche molecules, such as OPN and JAG1 in the BM of th3 mice. Interestingly, these molecules are directly regulated by PTH in OBs and MSCs [49], and their reduction leads to a loss of HSCs quiescence [50]. PTH is a key player of calcium and phosphate homeostasis, regulating bone remodeling and HSC maintenance via its specific receptor on BM stromal cells [49]. Aprile and colleagues demonstrated that the downregulation of PTH- JAG1-Notch1 axis and OPN in BThal leads to defective stromal BM niche with impaired bone deposition and defective crosstalk between osteolineage cells and HSCs [114]. These findings were also validated in patients’ samples.

Since MSCs also produce JAG1, the authors analyzed this population in th3 mice. MSCs are characterized by decreased frequency and lower expression of Jag1 as compared to wt controls [114]. Consistently, MSCs from BThal patients showed reduced frequency and clonogenic potential, lower proliferation rate, impaired differentiation potential, and a reduced capacity to support HSPCs [127].

**SCD.** Individuals suffering from SCD experience acute and chronic bone pain caused by occlusive events within the tissue vasculature that result in ischemia, necrosis, and organ degeneration [34]. However, the pathophysiology of bone defects is still under investigated. Recent studies have suggested that environmental stimuli, such as inflammation, may influence the osteoporotic-like phenotype observed in SCD bone [128,129]. Because of the interactions between SS-RBCs and vascular endothelium, SCD patients display abnormally high concentrations of inflammatory molecules, especially IL-6, IL-8, and TNF-α. In healthy conditions, inflammation plays a crucial role in regulating bone remodeling; however, the chronic inflammation localized within the bone microcirculation may prolong OC activity via the upregulation of RANKL [130,131]. Micro-CT image analysis of transgenic SCD mice showed altered bone microarchitecture with fewer trabeculae and deteriorated structure, indicating progressive damage of SCD bone tissue [132]. Dalle Carbonare et al. reported that recurrent hypoxia/reperfusion events, mimicking acute VOCs, activate osteoclastogenesis and bone turnover in SCD mice, with upregulation of the pro-resorptive cytokine IL-6 and suppression of osteogenic lineage markers, such as *Runx2* and *Sparc* [133]. The administration of zoledronic acid, a potent inhibitor of osteoclastogenesis and OC activity, ameliorated bone impairment and promoted osteogenic lineage. These data supported the view that bone disease in SCD is related to the biased coordination of remodeling signals towards bone absorption. In addition, the reduced OB recruitment and the increased OC activity are induced by local hypoxia, oxidative stress, and the release of IL-6. Furthermore, IO due to chronic transfusions was reported to increase bone resorption and impair the trabecular microarchitecture in SCD [134].

Human MSCs derived from SCD BM were found to have altered expression of SCF and CXCL12, but showed normal in vitro functionality [135]. However, recent work by Tang et al. reported a reduced frequency of MSCs in the BM of SCD mice, ROS accumulation, and a decreased adipogenic and osteogenic differentiation potential, also suggesting impaired MSC functional properties [115]. Gene expression profiling revealed a decreased transcription of key niche molecules, such as *Opn*, as well as vascular cell adhesion to protein 1 (*Vcam1*)*, Angpt1*, *Scf*, and *Cxcl12*, associated with an impaired ability to maintain HSCs in vitro and in vivo. These data are in line with increased HSC mobilization and reduced engraftment upon transplantation. Treatment with NAC and transfusions reduced MSC oxidative stress and improved the crosstalk between SCD MSCs and HSCs. Activation of TLR4 by hemolysis contributed to SCD MSC dysfunction.

Alterations in the BM vasculature were reported to be critical for SCD hematopoiesis too. Park et al. demonstrated that SCD mice have a disorganized BM vascular network with increased numbers of highly tortuous arterioles and fragmented sinusoidal vessels [136]. In SCD, slow RBC flow and vaso-occlusions diminish local oxygen availability in the BM cavity and increase ROS production. Elevated levels of HIF-1α were found, triggering an enhanced neovascularization. Transplantation of BM cells from SCD mice into wt recipients recapitulated the SCD vascular phenotype by increasing HIF-1α signaling in normal mice. Conversely, blood transfusions completely reversed the altered vascular network, highlighting the plasticity of the BM vascular niche.

### 4.3. Hematopoietic and Soluble Niche Factors

**BThal.** Data on BThal mice indicate that multi-factorial alterations in the BM niche can impair HSC self-renewal and repopulating capacity. During the analysis of stromal components and soluble factors of the BM microenvironment, high systemic and BM local levels of FGF23 were detected [137]. FGF23 is a negative regulator of bone metabolism and PTH secretion [138], mainly produced by bone and erythroid cells in response to the anemia-related factor EPO [139]. The enhanced activation of FGF23 signaling has been proposed as the mechanism underlying bone disease and low PTH levels in th3 mice, negatively impacting the functional crosstalk between HSCs and the stromal niche [137].

In addition to the BM stroma, altered levels of multiple local and systemic factors were found, including SCF, ANGPT1, and CXCL12 [114], as well as a reduction in serum TPO [140]. Since TPO is a key regulator of both HSCs [53,141] and MKs [73,142], the TPO defect can have a dual role on BThal HSCs and BM microenvironments, thus contributing to the impaired HSC–niche crosstalk. Moreover, the condition of chronically reduced TPO stimulation in BThal is consistent with reported results of higher cycling activity of HSCs in the absence of TPO [141], and effectively correlates with data of low HSC quiescence in th3 mice [114]. Low TPO also impacts MK maturation and their downregulated expression of niche molecules in th3 mice [140].

By focusing on other hematopoietic populations of the BM niche, different BM resident Mφs have been reported to indirectly regulate HSC retention by acting on niche stromal cells [143]. Thus, the dissection of different populations of BM Mφs in BThal can contribute to the enhanced proliferation, increased mobilization, and reduced repopulating potential of th3 HSCs. Since BThal is characterized by IE with reduced erythroid terminal differentiation and expansion of the BM erythroid precursors, as expected, the frequency of erythroblastic island Mφs, essential for erythroblast survival and maturation, was significantly increased (unpublished data). BThal neutrophils were reported to display aberrant maturation and defective effector functions [144]. Reduced BM Mφ–neutrophil interactions can play a role in BThal HSC mobilization, through the indirect effect on the production of CXCL12 retention molecule by the BM stromal niche [82]. Preliminary data from the th3 mouse model showed an imbalanced polarization towards the M1 phenotype and a reduced neutrophil clearance by BThal BM Mφs, suggesting a potentially negative effect on HSCs (unpublished data).

**SCD.** In the complexity of SCD pathophysiology, many hematopoietic populations and soluble factors potentially involved in the regulation of the BM microenvironment homeostasis are altered [145]. However, their direct contribution to the HSC niche is still completely unexplored.

Among the more studied populations, neutrophils of SCD patients and mice were activated by the increased production of ROS [146]. They contribute to SCD pathogenesis by capturing circulating SS-RBCs, inducing VOCs, and secreting inflammatory cytokines [147]. Free heme induces neutrophil extracellular trap (NET) formation by activated neutrophils, significantly contributing to SCD pathogenesis [148]. In SCD mice, the aged neutrophil population is expanded and positively correlates with adhesion and interactions with RBCs. Neutrophil ageing is regulated by the microbiome [149] and neutrophil clearance by BM was reported to modulate the HSC niche [82]. Thus, the involvement of SCD neutrophils can be hypothesized in the regulation of the BM microenvironment.

Furthermore, platelets and monocytes in SCD were reported to have an activated phenotype with an active role in VOC pathogenesis, promoting the inflammation state associated with the disease [150,151]. SCD indeed have long been recognized as a chronic inflammatory disease, and, during infection or systemic inflammation, HSCs were reported to respond directly to inflammatory triggers [152], leading to their activation, expansion, and enhanced myeloid differentiation [153]. Furthermore, increased circulating heme and iron, i.e., hallmarks of SCD, were shown to induce Mφ phenotypic switching toward an M1 proinflammatory phenotype [154], which has been reported to negatively regulate HSC maintenance [81]. Whether BM cell populations and HSC function are altered in SCD in response to pro-inflammatory stimuli still needs to be explored.

### 4.4. The Role of IO in the BThal BM Niche

IO, associated with IE and therapeutic blood transfusions, is a key element of BThal pathophysiology. Despite improvements in chelation therapies over the past few years, the BThal BM niche accumulates a high content of iron. The direct impact of IO on BThal HSCs remains poorly characterized [155]. Data on BThal th3 mice showed a positive enrichment of genes involved in iron homeostasis and significantly high levels of free reactive iron in HSCs, which correlate with increased ROS content (unpublished data).

Recently, Crippa et al. demonstrated that IO negatively affects BM MSCs in TDT patients [127]. The in vitro exposure of BThal MSCs to increasing doses of iron revealed an upregulation of iron transporters, such as *ZIP14*, *ZIP18*, transferrin receptor 1 (*TFR1*), and ferritin, thus suggesting that BThal MSCs can uptake and store iron. These findings are corroborated by the direct assessment of iron content in BThal MSCs using Perl’s staining. BThal MSCs display high ROS levels, as a result of the impaired antioxidant response, which correlates with a significant pauperization of the most primitive CD146^+^ CD271^+^ MSC pool. BThal MSCs showed reduced clonogenic capacity, lower proliferation rate, early cell cycle arrest, and impaired differentiation potential into adipocytes and bone. In addition, they express lower levels of hematopoietic supportive factors, such as SCF, *CXCL12*, cadherin 2 (*CDH2*), *VCAM*, *ANGPT1*, vascular endothelial growth factor A (*VEGFA*), *IL-6*, and *FGF2*. Therefore, they fail to attract and expand HSCs in transwell migration assays and 2D co-culture experiments. Consistently, the in vivo transplantation of CD34^+^ HSPCs, along with BThal MSCs, revealed a reduced hematopoietic engraftment upon xenotransplantation in NSG mice. Finally, the authors developed a humanized ossicle model, consisting of gelatin scaffold pre-seeded with MSCs, ECs, and CD34^+^ HSPCs, to test the ability of BThal MSCs to form a proper BM niche in vivo. Transplantation of the humanized ossicle into NSG mice showed a delay in the formation of bone and vessels, as well as a reduced number of human CD45^+^ hematopoietic cells ossicles derived from BThal MSCs.

Strikingly, treatment with the iron chelator deferoxamine (DFO) in the presence of iron decreased the expression of iron transporters, potentiated the antioxidant defense system in BThal MSCs, and rescued the expression of the hematopoietic supportive factors [127]. Therefore, treatment with chelating agents or antioxidants can represent a therapeutic strategy to ameliorate BThal MSC supportive capacity, thus potentially improving the transplantation outcome.

**Figure 1:**
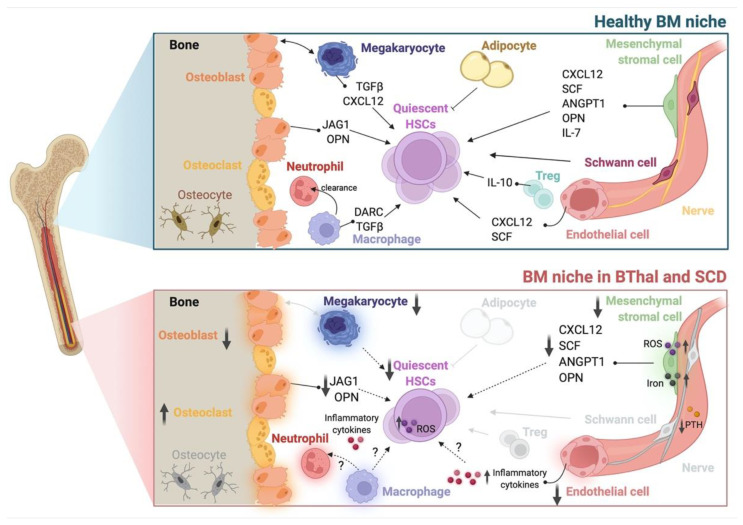
The BM HSC niche in healthy conditions and in BThal and SCD. Schematic representation of the adult BM niche in homeostasis, showing different stromal (osteoblasts, mesenchymal stromal cells, endothelial cells, Schwann cells, nerve fibers, and adipocytes) and hematopoietic (megakaryocytes, osteoclasts, macrophages, neutrophils, and Tregs) cell types and niche factors that regulate HSC quiescence and function (**upper panel**). The BM niche in BThal and SCD shows alterations in BM osteolineage cells, mesenchymal stromal cells, endothelial cells, and megakaryocytes, causing the reduced production of niche molecules supporting HSC activity. The accumulation of ROS, iron, and inflammatory cytokines contributes to the impairment of HSC maintenance (**bottom panel**). Created with BioRender.com.

Overall, these findings highlight previously unexplored multifactorial alterations of BM components in the biocomplexity of BThal and SCD (Table 1 and Figure 1). Elucidating the overriding players and the functional interconnections between stromal and hematopoietic alterations of the BM HSC niche will pave the way towards potential combined therapies, not only targeting the genetic defect, but also HSC and the BM microenvironment, in order to improve HSC transplantation and gene therapy approaches.

## 5. Targeting the HSC Niche in BThal and SCD

The correction of genetic defects in BThal and SCD is achieved by HSCT from normal donors or by experimental gene therapy with the transplantation of autologous genetically modified cells. In both settings, the transplanted HSCs and the recipient BM niche are central elements.

In comparison to other indications for allogeneic HSCT, there is an unexplained increased risk of graft failure, including cases of late rejection and mixed chimerism [156,157]. Especially in the autologous gene therapy HSCT, where both the donor HSCs and the recipient BM niche are diseased, the additive effect of an impaired HSC function and a defective supporting activity by the BM niche components, worsened by age and disease progression, can hamper the engraftment of genetically modified HSCs. The clinical benefits of gene therapy are dependent on several factors, including the patient’s clinical condition, the extent of genetic modification, the dose and quality of the engineered engrafting cells, and the status of the recipient BM niche [37,44]. It is reasonable to expect a negative effect of the BM microenvironment on HSC function, leading to potentially impaired reconstitution and premature exhaustion. In recent gene therapy trials for hemoglobinopathies, some cases of absence of clinical benefit, despite the occurrence of early hematopoietic engraftment, have been reported [9,10,11]. Low levels of genetically modified HSPCs in patients with a lack of clinical benefit can lead to impaired HSC function, as well as the defective supporting activity by niche components, although the root causes have not been clarified yet. Variability in terms of HSC transduction and in vivo reconstitution poses limitations for clinical outcome, and the status of the BM microenvironment influences the quality of HSCs harvested for genetic engineering, as well as their engraftment and reconstitution capacity once transplanted. Therefore, the comprehension and targeting of key mechanisms influencing HSC potential offer new avenues to improve gene therapy and develop combined transplantation approaches.

Moreover, myeloablative conditioning is required in order to obtain high levels of donor grafts upon HSCT, also in the autologous gene therapy setting, when reduced intensity conditioning is preferred. The effects of reduction in hematopoietic BM populations and the potential damages to the BM stroma are largely unknown and can have an impact on the supportive capacity of the BM niche. Recent advances in biological conditioning strategies by antibody–drug conjugates showed better preservation in the BM architecture. In this view, more studies are needed to elucidate the influence of conditioning on HSC niche activity.

**Figure 2:**
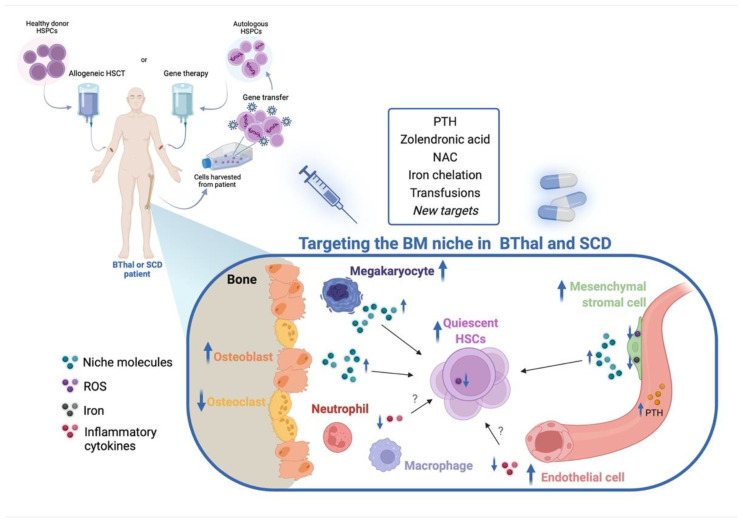
Targeting of the BM niche in BThal and SCD. Administration of treatments targeting the altered HSC niche components can be developed in combination with allogeneic or autologous gene therapy HSCT to restore the BM microenvironment. This may ameliorate the quality of patient-derived HSCs that are harvested and manipulated in gene therapy settings and, at the same time, make the BM niche more permissive for the engraftment of donor cells, both in the allogeneic and the autologous HSCT. Created with BioRender.com.

Here, we reported proof of concept studies aimed to rescue the identified alterations of HSCs and BM niche components in BThal and SCD (Figure 2). Although several works described alterations in SCD BM niche components, functional studies dissecting the defective interactions between HSC and BM microenvironments are missing. Future investigation can provide new therapeutic targets to restore the HSC–niche crosstalk.

### 5.1. Targeting the BM Stromal Niche

The pioneering work by Aprile and colleagues challenged the paradigm of BThal, as a disorder confined to erythropoiesis, demonstrating that HSCs are impaired in BThal due to a defective crosstalk with the BM microenvironment because of secondary complications to the primary genetic defect [114]. Most importantly, these data emphasized the reversibility of HSC features in stressed conditions since the authors showed that the defect can be rescued by the administration of PTH and the correction of the BM stromal niche. Indeed, in vivo PTH treatment to th3 mice normalized BMD, MSC frequency, and the expression of OPN and JAG1 by the BM stromal niche, thus resulting in the restoration of HSC function. In BThal patients, treatment with PTH before HSC harvest for gene therapy can protect HSC function and improve long-term engraftment. Therapeutic targeting of the HSC niche by PTH stimulation has provided evidence of an effective strategy to stimulate the HSC pool and increase its engraftment upon transplantation [158], although subsequent studies using PTH treatment after cord blood transplantation in a heterogenous group of patients reported disappointing results [159]. The balance between the anabolic and catabolic effects of PTH needs careful clinical evaluation. Thus, more tunable molecules targeting the BThal stromal niche alterations should be explored and, in this view, investigation of mechanisms acting upstream the bone and PTH defects could be a promising option. Encouraging results highlighted the efficacy of the FGF23 inhibition strategy to correct bone mineralization and deposition and rescue the impaired HSC–niche crosstalk in BThal [137].

On the same line, approaches to correct secondary defects with a potential impact on the BM niche were exploited in SCD. The administration of zoledronic acid, a bisphosphonate drug acting as an inhibitor of osteoclastogenesis and OC activity, ameliorated bone defects and promoted osteogenic lineage in SCD mice [133]. However, the positive effect on the HSC niche remains unexplored.

Other potential targeting strategies are those which aim to target primary defects, i.e., anemia. Blood transfusions are the mainstay of treatment for BThal and SCD and can also ameliorate secondary complications. Park et al. demonstrated that a 6-week transfusion regimen completely reversed the abnormal SCD BM vasculature by reducing proangiogenic mediators, such as VEGF-A, ANG1, and ANG2, as well as markers of inflammatory vascular activation [71]. Transfusions were demonstrated to also alleviate SCD MSC disfunction [115]. Upon transfusion, SCD MSCs showed decreased ROS content and enhanced expression of *Scf* and *Cxcl12*, correlating with increased HSC frequency. Similarly, NAC antioxidant treatment improved SCD MSC ability to maintain HSCs [115].

### 5.2. Targeting IO and Other Stress Signals

IO due to hemolysis and IE are common hallmarks of BThal and SCD, although damage caused by IO to the BM microenvironment is largely unknown.

IO in BThal BM was reported to have a negative impact on patient-derived MSCs, reducing their frequency, differentiation ability, and hematopoietic supportive capacity [127]. Thus, targeting the BM niche to reduce iron-induced oxidative stress can ameliorate HSCT outcomes. Crippa et al. suggested looking for potential associations between the BM state and the risk of graft failure in BThal patients undergoing both allogeneic or gene therapy HSCT, by correlating the levels of BM IO with data of the transplant outcome. Optimizing current iron chelation therapy can ameliorate MSC status, with potential effects on other BM niche populations and HSCs.

Increased circulating heme and iron is also a hallmark of SCD pathophysiology. Heme, indeed, acts as a proinflammatory molecule that activates ECs, neutrophils, and Mφs. Hemopexin, an extracellular scavenging system that binds heme, was reported to revert heme-induced M1 switching of Mφs in SCD mice [154] and to reduce endothelial toxicity caused by heme in SCD and BThal models [160]. Whether heme and hemopexin treatment can play a role in the SCD BM microenvironment is still unknown.

Antioxidant and anti-inflammatory treatments can be effective in restoring the BM niche and HSC functions impaired by oxidative stress and inflammation, respectively. For example, NAC treatment was demonstrated to improve the engraftment capacity of HSPCs in a mouse model of SCD [123].

## 6. Conclusions and Future Perspectives

Over the last few decades, research on BThal and SCD has been mostly focused on erythropoiesis, leaving the study of the BM microenvironment and HSCs underexplored. Recent studies demonstrated that HSC and BM niche components are altered in BThal and SCD, and that targeting the identified defects can rescue the impaired HSC–BM niche crosstalk. Thus, supportive therapies which aimed to ameliorate the BM niche and preserve long-term HSC function need to be pursued and developed. In this view, the combination of autologous gene therapy with niche targeting has the potential to improve therapeutic outcomes. Treatment pre- and post-HSCT can refurbish bone and HSC niche before HSC harvest and promote early engraftment after gene therapy.

Since BThal and SCD represent the most widespread monogenic diseases worldwide, the impact of novel combined treatments also targeting the BM niche could be relevant, leading to significant advances in hematology. An investigation of the BM alteration to develop potential new pharmacological treatments can represent an effective bridge between basic research and clinical translation from genetic Hb disorders to other diseases associated with erythropoietic stress.

The works reviewed here open up new questions and point out the need for a deeper understanding of HSC biology and interactions with the BM niche in diseases. These studies on BThal and SCD mouse models and patient-derived samples pave the way towards a novel concept—the complementary investigation of hematopoiesis, not only in steady state, acute stress, and malignancies, but also in non-malignant hematological disorders, with a potential impact on HSCT, i.e., the only definitive cure for hemoglobinopathies.

Further investigation will open up new avenues in this line of research.

## Figures and Tables

**Table 1:** Altered BM niche populations in BThal and SCD. Alterations are shown as increased (⇑) or decreased (⇓) levels of specific features in BM niche populations.

Cell Population	Disease	Species	Alterations	References
HSC	BThal	mouse	⇓ number ⇓ quiescence ⇓ stemness ⇓ reconstitution capacity ⇑ response to stress	[114]
		human	⇓ frequency ⇓ quiescence ⇓ stemness ⇑ response to stress (HSPC)	[114,121,122]
	SCD	mouse	⇓ frequency ⇑ ROS ⇑ DNA damage ⇓ quiescence (HSPC) ⇑ mobilization (HSPC)	[115,123,124]
		human	⇓ frequency ⇑ mobilization	[121,125]
Osteolineage cell	BThal	mouse	⇓ BMD ⇓ systemic PTH ⇓ OB activity ⇓ niche molecules ⇑ FGF23	[114,126,137]
		human	⇓ niche molecules	[114]
	SCD	mouse	⇓ bone microarchitecture	[132]
			⇑ osteoclastogenesis ⇓ osteogenic factors	[133]
MSC	BThal	mouse	⇓ frequency ⇓ niche molecules	[114]
		human	⇓ frequency ⇓ osteogenic and adipogenic potential ⇑ ROS ⇑ iron content ⇓ niche molecules ⇓ HSPC maintenance	[127]
	SCD	mouse	⇓ frequency ⇑ ROS ⇓ osteogenic and adipogenic potential ⇓ niche molecules ⇓ HSC maintenance	[115]
		human	⇓ niche molecules	[135]
EC	SCD	mouse	altered BM vasculature ⇑ inflammatory cytokines	[136]
MK	BThal	mouse	⇓ systemic TPO ⇓ maturation ⇓ niche molecules	[140]
Neutrophil	BThal	mouse	altered maturation	[144]

## Data Availability

Not applicable.
